# Reducing the stigma surrounding opioid use disorder: evaluating an opioid overdose prevention training program applied to a diverse population

**DOI:** 10.1186/s12954-022-00589-6

**Published:** 2022-01-16

**Authors:** Nicholas Alexander Bascou, Benjamin Haslund-Gourley, Katrina Amber-Monta, Kyle Samson, Nathaniel Goss, Dakota Meredith, Andrew Friedman, Andrew Needleman, Vishnu K. Kumar, Bradford D. Fischer

**Affiliations:** 1 Cooper Medical School of Rowan University, Camden, NJ USA; 2 Drexel University College of Medicine, Philadelphia, PA USA

## Abstract

**Background:**

The opioid epidemic is a rapidly growing public health concern in the USA, as the number of overdose deaths continues to increase each year. One strategy for combating the rising number of overdoses is through opioid overdose prevention programs (OOPPs).

**Objective:**

To evaluate the effectiveness of an innovative OOPP, with changes in knowledge and attitudes serving as the primary outcome measures.

**Methods:**

The OOPP was developed by a group of medical students under guidance from faculty advisors. Training sessions focused on understanding stigmatizing factors of opioid use disorder (OUD), as well as protocols for opioid overdose reversal through naloxone administration. Pre- and post-surveys were partially adapted from the opioid overdose attitudes and knowledge scales and administered to all participants. Paired t-tests were conducted to assess differences between pre- and post-surveys.

**Results:**

A total of 440 individuals participated in the training; 381 completed all or the majority of the survey. Participants came from a diverse set of backgrounds, ages, and experiences. All three knowledge questions showed significant improvements. For attitude questions, significant improvements were found in all three questions evaluating confidence, two of three questions assessing attitudes towards overdose reversal, and four of five questions evaluating stigma and attitudes towards individuals with OUD.

**Conclusions:**

Our innovative OOPP was effective not only in increasing knowledge but also in improving attitudes towards overdose reversal and reducing stigma towards individuals with OUD. Given the strong improvements in attitudes towards those with OUD, efforts should be made to incorporate the unique focus on biopsychosocial and sociohistorical components into future OOPPs.

**Supplementary Information:**

The online version contains supplementary material available at 10.1186/s12954-022-00589-6.

## Background

The opioid epidemic is a rapidly growing public health crisis in the USA, with over 500,000 deaths attributed to opioid overdoses since the mid 1990s. Essentially, the public health crisis began in large part from the overprescription of pharmaceutical analgesics, which have gradually been replaced by more potent and potentially lethal black market drugs, such as heroin and fentanyl [1]. In 2017 alone, 47,506 deaths were a direct result of opioid overdose (a notable percentage of victims were also positive for consumption of other drugs, such as alcohol or benzodiazepines) [2]. In addition, there has been a trend towards increasing death rates over the past 2 decades, as opioid-related overdose deaths per 100,000 persons rose from 2.20 to 13.21 between 2000 and 2017 [3]. Since the Covid-19 pandemic, this devastating trend has accelerated exponentially, with opioid-related overdose deaths recorded in the 12 months leading up to April 2021 reported as 75,673, a staggering increase of 28.5% compared to the previous 12 month time interval [4].

One method to mitigate the decades-long trend of increasing opioid overdose deaths may be accomplished through naloxone and opioid overdose prevention programs (OOPPs). The World Health Organization (WHO) recommends “people likely to witness an opioid overdose should have access to naloxone and be instructed in its administration” [5]. To date, there have been a myriad of studies published evaluating the efficacy of naloxone and OOPPs, some of which are briefly reviewed here (Table 1) [6–36].

**Table 1:** Summary of notable studies on OOPPs that have been published previously

References	Participants	# of participants w/post-training data	Setting	Assessment method	Knowledge and/or confidence	Attitudes toward OUD	Attitudes towards naloxone
Green et al. [6]	Individuals w/OUD	62	In-person	Comparing trained versus nontrained ability to recognize OD	Improved	NA	NA
Galea et al. [7]	Individuals w/OUD	25	In-person	Pre- versus post-training responses to OD	Improved	NA	NA
Piper et al. [8]	Individuals w/OUD	120	In-person	Post- OD survey	No baseline	NA	NA
Strang et al. [9]	Individuals w/OUD	186	In-person	Pre- versus post-training survey versus 3-month follow-up	Improved	NA	NA
Doe-Simkins et al. [10]	Individuals w/OUD	57	In-person	Qualitative descriptions of post-training responses to OD	NA	NA	NA
Gaston et al. [11]	Individuals w/OUD	70	In-person	Pre- versus post-training survey versus 3 month follow-up versus 6-month follow-up	Improved	NA	NA
Tobin et al. [12]	Individuals w/OUD	43	In-person	Pre- versus post-training survey	Improved	NA	NA
Enteen et al. [13]	Individuals w/OUD	1942	In-person	Number of reported OD reversals	NA	NA	NA
McAuley et al. [14]	Individuals w/OUD	19	In-person	Pre- versus post training survey	Improved	NA	NA
Wagner et al. [15]	Individuals w/OUD	47	In-person	Pre- versus post training survey versus 3-month follow-up; Response to OD	Improved	NA	NA
Bennett et al. [16]	Individuals w/OUD	89	In-person	Qualitative descriptions of post-training responses to OD	NA	NA	NA
Bennett et al. [17]	Individuals w/OUD	521	In-person	Pre- versus post-training survey	Improved	NA	NA
Yokell et al. [18]	Mixed population	10	In-person	Qualitative descriptions of post-training responses to OD	NA	NA	NA
Walley et al. [19]	Individuals w/OUD	62	In-person	Descriptions of post-training responses to OD	NA	NA	NA
Walley et al. [20]	Mixed population	212	In-person	Descriptions of post-training responses to OD	NA	NA	NA
Dietze et al. [21]	Mixed population	683	In-person	Pre- versus post-training survey	Improved*	NA	NA
Kwon et al. [22]	Pharmacy students	56	In-person	Pre- versus post-training survey	Improved	NA	NA
Zhang et al. [23]	EMTs	117	In-person	Pre- versus post training survey	Improved	No change	Improved
Wagner et al. [24]	Police officers	81	In-person	Pre- versus post training survey	Improved	No change	Improved
Williams et al. [25]	Family members	123	In-person	Post-surveys from trained versus non-trained	Improved	Improved	Improved
Lewis et al. [26]	Mixed population	113	In-person	Pre- versus post survey versus 8 month and 12 month interview	NA	NA	Improved
Ray et al. [27]	Police officers	117	In-person	Post-training survey	No baseline	No baseline	No baseline
Ashrafioun et al. [28]	Mixed population	428	In-person	Pre- versus post-training survey	Improved	NA	NA
Berland et al. [29]	Medical students	120	In-person	Pre- versus post-training survey	Improved	No change	NA
Dahlem et al. [30]	Police officers	114	In-person	Pre- versus post-training survey versus 1 year follow-up	Improved	NA	NA
Hill et al. [31]	Pharmacy students	94	In-person	Post-surveys from trained versus non-trained	Improved	NA	Improved
Bachyryz et al. [32]	Pharmacy students	141	In-person	Pre- versus post-training survey	Improved	NA	Improved
Goss et al. [36]	Medical students	150	In-person and online	Pre- versus post-training survey	Improved	Improved*	Improved
Moses et al. [33]	Medical students	190	In-person	Pre- versus post-training survey	Improved	NA	Improved
Halmo et al. [34]	Social work students	33	In-person	Pre versus post-training survey	Improved	NA	Improved

While it displays many earlier studies, the table is not comprehensive. NA = Not assessed; THN = Take home naloxone; OD = overdose; Improved* = Only improvements in the minority of questions

Although the concept of OOPPs was first introduced in 1996, formal evaluations of training and distribution programs did not begin until the 2000s [35]. At the onset, the overwhelming majority of OOPPs were directed towards training individuals suffering from opioid use disorder (OUD), where participants would receive naloxone at the end of the session, coining the name take-home naloxone (THN). Initial studies on THN provided invaluable information focusing primarily on whether the programs led to reversals of opioid overdose, although there was generally no evaluation on the program’s effects on knowledge or on participants’ level of comfort to intervene [7, 10]. Given these limitations, investigators soon began to shift the paradigm, focusing on study designs favoring pre- versus post-training survey comparisons. These later studies provided convincing evidence that OOPPs are highly effective in increasing knowledge, competency, and confidence in the use of naloxone [9, 11, 12]. Subsequently, the next step in the evolution of OOPPs moved towards educating populations outside of those with OUD, as well as supplementing evaluation of participant knowledge with an assessment of participant attitudes towards overdose reversal and individuals suffering from OUD.

One of the most notable studies to investigate changes in attitudes included 123 family members, all of whom were related to an individual deceased due to opioid overdose. Here, subjects were divided into trained and untrained groups, and improvements were found in both knowledge and attitudes towards overdose reversal and OUD within the trained group. Of interest, this was the first study to utilize the now standard opioid overdose and attitudes scale (OOAS) and is one of two studies to date that has demonstrated significant improvements in attitudes towards individuals with OUD [25, 36]. The second study was recently published by our group and involved a comparative analysis of online versus in-person training in a population of medical students. While the study found no significant differences between training modalities, it did find improvements in attitudes towards overdose reversal, as well as limited, albeit encouraging, improvements in attitudes towards individuals with OUD [25, 36]. Unfortunately, most other studies have failed to find any improvements in attitudes towards individuals with OUD. For example, a recent study conducted using a sample of 120 medical students at the New York University School of Medicine extensively analyzed changes in knowledge and attitudes towards OUD using [29, 37]. While the study found improvements in knowledge, they did not detect any statistically significant change in attitudes [29]. Various studies conducted on other demographic populations, such as police officers and pharmacy students, have yielded similar outcomes (Table 1).

In summary, these prior studies have demonstrated promising results by increasing trainees’ knowledge and competency in reversing opioid overdose. However, all aforementioned studies were pervaded by one or more of the following limitations: (a) relatively small sample size; (b) simplicity of pre/post-surveys; (c) homogenous sample population with potential for inherent selection bias; and/or (d) no assessment for changes in attitude regarding OUD. Furthermore, there is a need for a study evaluating a large sample of diverse individuals through the implementation of a detailed pre/post-survey that sufficiently assesses for both changes in competency and shifts in attitudes. Although a potentially difficult task, it is important to note that assessing attitudes is crucial since stigma and negative attitudes towards people with OUDs have been shown to undermine secondary prevention responses [38]. We believe advancements can be made on this front by designing OOPPs that provide not only training in the use of naloxone but also combat the negative stigmas, biases, and legislative regulations that are responsible for perpetuating the opioid epidemic, since current approaches continue to fall short in reducing the devastation caused by OUD.

Thus, the purpose of this project is to address the gap in the literature via evaluation of our unique Opioid Overdose Awareness and Reversal Training (OOART). In short, the objectives of our OOART are to: (1) increase the public’s awareness of the large-scale devastation caused by opioids, (2) enable participants to view OUD from the perspective of an individual suffering from OUD in order to increase empathy and reduce stigma, and (3) increase the number of individuals versed in the use of naloxone and overdose reversal. We hypothesize that participation in the OOART training sessions will produce significant improvements in the primary outcomes, which are improving competency and attitudes regarding the use of naloxone in overdose reversal and OUD as a whole. Our ultimate goal is that this study will represent a critical step towards slowing the rate of opioid-related overdose death by summoning public awareness of the deeply ingrained biases and stigma surrounding the epidemic.

## Materials and methods

### Development of OOART

The OOART was developed by a group of medical students at Drexel University School of Medicine, with assistance from faculty advisors and members of the Philadelphia Department of Public Health. Of note, multiple individuals key in the design and implementation of the study have lived-experience with OUD. In addition to providing practical knowledge of naloxone administration, our OOART provides unique insights into the sociohistorical development of the opioid epidemic and biopsychosocial considerations pertinent to those suffering from OUD. In short, the OOART is divided into two parts: a PowerPoint presentation and an overdose simulation. The PowerPoint was split into seven sections: (1) Opioid Basics, (2) Introduction to the Opioid Epidemic, (3) A Brief History and the Aftermath, (4) The Experience of OUD, (5) Disparities in the Opioid Epidemic, (6) OUD Treatment and Harm Reduction as a Tool, and (7) Overdose Reversal, Naloxone Administration, and Post-Reversal Care. A breakdown of the presentation has been described previously [36], with a more detailed outline of the discussion topics depicted in Additional file 1: Appendix 1. Following each presentation, two trainers simulated an overdose situation, where one acted as the individual who overdosed and the other as the ‘Good Samaritan’ performing the overdose reversal. Afterward, participants were encouraged to practice on each other or a CPR manikin using a practice nasal naloxone applicator (with the exception of online trainings, where this was not possible). Each training concluded with an individual who has personally suffered from OUD discussing their lived experiences with the group.

Readers should note that although the nasal form of naloxone, Narcan, was used in these training sessions, for consistency, we will refer to the opioid antagonist only as its generic name throughout the remainder of the paper.

### Survey

Surveys were administered both prior to training and after training. The pre-survey was utilized to collect demographic information, including age, gender, and employment status. It also questioned participants if they have ever been previously trained in the use of naloxone, if they have ever witnessed an overdose, if they have ever administered naloxone, and whether or not they are currently carrying naloxone. The remaining questions were identical between the pre- and post-survey and were used to obtain data on competency and attitudes.

In terms of attitudes, the survey included 11 questions. These questions were further divided into three subsections: (a) “Attitudes Towards Naloxone and Overdose Reversal,” (b) “Attitudes Towards Individuals with OUD”, and (c) “Self-Confidence in Using Naloxone and Handling an Overdose.” Given that trainees engaged in sessions up to 3 h in length, we found in pilot studies that using the original 28 question OOAS survey and the 15 OOKS (totalling 43 questions) lead to a majority of incomplete post-surveys, which necessitated shortening of the survey length. Furthermore, our modified survey included six questions from the original OOAS that were deemed to have the least amount of redundancy and overlap with one another [39]. These six were placed in the category of either “Attitudes Towards Naloxone and Overdose Reversal” or “Self-Confidence in Using Naloxone and Handling an Overdose.” In addition, because we found that the original OOAS lacked any substantial questions attempting to evaluate stigma or participants’ biases and attitudes towards those with OUD, we devised five more questions that were specifically designed to address these issues. We then grouped these five into the category “attitudes towards individuals with OUD.” The exact wording and categorization of the questions are presented in Table 2. All attitudes questions were scored on a 5-point Likert Scale (Completely Disagree = 1; Disagree = 2; Unsure = 3; Agree = 4; Completely Agree = 5). To assess competency, the survey included 3 multiple-choice fact-based questions that were adapted and shortened from the Opioid Overdose Knowledge Scale [39]. The competency questions were scored as either “1” for correct or as “0” for incorrect. This allowed for comparison between individual questions and between overall percent correct (Table 2).

**Table 2:** Results of pre- and post-surveys used to assess attitudes, confidence, and competency

Question	Pre-test	Post-test	*p* value
*Attitudes towards narcan usage and overdose reversal*			
If someone overdoses, I want to be able to help them? (*N* = 381)	Mean = 4.90 (SD: 0.38)	Mean = 4.93 (SD: 0.25)	*p* = 0.0897
Everyone should learn how to use and carry naloxone (*N* = 380)	Mean = 4.34 (SD:0.75)	Mean = 4.64 (SD: 0.64)	*p* < 0.0001***
I will do whatever is necessary to save someone's life in an overdose situation (*N* = 380)	Mean = 4.66 (SD: 0.53)	Mean = 4.74 (SD: 0.47)	*p* = 0.0016**
*Attitudes towards individuals with OUD*			
It is understandable why those who use drugs and experience withdrawal symptoms may use drugs daily (*N* = 376)	Mean = 4.44 (SD: 0.73)	Mean = 4.68 (SD: 0.57)	*p* < 0.0001***
We need to provide ways to keep people alive and minimize the harms associated w/ drug use to effectively deal w/ the opioid epidemic (*N* = 381)	Mean = 4.76 (SD: 0.46)	Mean = 4.85 (SD: 0.38)	*p* < 0.0001***
People often start using opiods, and find it hard to quit due to a lack of willpower and discipline (*N* = 379)	Mean = 2.25 (SD: 1.12)	Mean = 2.07 (SD: 1.24)	*p* = 0.0341*
It is understandable that many people are not ready, willing, or able to get treatment for substance use disorder (*N* = 379)	Mean: 4.23 (SD: 0.76)	Mean = 4.55 (SD: 0.65)	*p* < 0.0001***
My attitudes toward people who use drugs, and how I think and talk about them, has nothing to do w/ their ability to seek or receive help (*N* = 368)	Mean: 3.43 (SD: 1.24)	Mean: 3.50 (SD: 1.48)	*p* = 0.3581
*Self-confidence in using naloxone and handling overdose*			
I would be afraid of doing something wrong in an overdose situation (*N* = 374)	Mean = 3.61 (SD: 1.11)	Mean = 2.52 (SD:1.11)	*p* < 0.0001***
If I saw an overdose, I would panic and not be able to help (*N* = 379)	Mean = 2.20 (SD: 0.87)	Mean = 1.74 (SD: 0.72)	*p* < 0.0001***
I would be able to deal effectively with an overdose (*N* = 381)	Mean = 3.43 (SD: 0.96)	Mean = 4.15 (SD: 0.80)	*p* < 0.0001***
*Knowledge/competency in using narcan and handling overdose*			
Percent (%) correct	Mean = 43% (SD: 15.58)	Mean = 94% (SD: 14.69)	*p* < 0.0001***

Mean and standard deviation (SD) are reported for all values. Statistically significance is designated by * for *p* < 0.05, ** for < 0.01, ***for *p* < 0.01

### Delivery of OOART

The majority of trainings were conducted in-person, although a portion were done through a live online platform. The online sessions were implemented during the early stages of the COVID-19 pandemic, when institution-wide restrictions on in-person meetings, made in-person meetings an impossibility. Nevertheless, all trainings were treated equally irrespective of platform, as Goss et al. [36] previously found no significant differences between in-person and online platforms. All trainings were conducted within Philadelphia or Southern New Jersey between October 2018 and October 2020. With the exception of regional bias, participants included individuals from a diverse set of backgrounds. These included medical students, physician assistant (PA) students, occupational therapy (OT) students, undergraduate students, police officers, internal medicine residents, members of a recovery program, and other community members. For in-person sessions, pre-surveys were distributed upon arrival, and participants were instructed to complete them prior to the presentation. At the end of the session, participants completed the post-survey and handed in both the pre- and post-surveys. Google surveys were used for online sessions in a similar manner. The length of the training sessions varied depending on the number of audience questions and extent of the concluding discussion but typically lasted 2–3 h.

As with many other OOPPs, we attempted to distribute naloxone to all participants and did so at the majority of trainings. However, distribution was not always possible depending on variations in funding. For events where we were unable to provide naloxone directly, we always highlighted at least two different ways that people could either purchase or obtain free doses through public health initiatives.

### Data analysis

Analysis for statistical differences between pre- and post-surveys was conducted using a paired t-test, and each survey question was analyzed independently. *p* values of < 0.05 were considered statistically significant. Descriptive statistics were also used to display the pre- and post-mean and standard deviation for each question. Figures were generated using GraphPad Prism Software Version 8.

### Recruitment and inclusion/exclusion criteria

For students, recruitment was done predominantly through emails and newsletters. Community trainings were conducted through collaboration with local businesses and recovery centers. Recruitment and collaboration in these instances were initiated through either word of mouth or the training team physically going door to door and inquiring if owners or managers were interested in hosting a training for their employees and/or clients. All participation was voluntary. Other than being a willing volunteer, there were no specific inclusion criteria, which enabled for recruitment of a diverse sample.

All demographic data were included in the results, regardless of whether the remainder of the survey was complete or incomplete. Criteria for analysis of attitude and competency questions were more stringent. Any participants who failed to complete more than half the pre- or post-survey questions were automatically excluded from the analysis. In addition, responses to individual questions were excluded from the analysis when participants answered the pre-training question but failed to respond to the corresponding post-training question or vice versa.

## Results

### Cohort characteristics

Between 2018 and 2020, there were 440 who underwent the OOART, with 381 participants completing both the pre- and post-training surveys. Of the 440 trainees, 372 participated in in-person sessions, and 68 participated in online sessions. Overall, an average survey response rate of 86.5% was observed. A brief review of the total participant demographics revealed a gender distribution with 36.8% males, 60.2% females, and 1.42% preferring not to say. The mean age was 26.1 years, with a range of 18–76 years. Occupation demographics revealed participants to be 54.1% medical students, 11.6% full-time employees (mix of healthcare related and non-healthcare related occupations), 7.95% OT students, 6.13% PA students, 6.13% undergraduates, 5.68% who did not disclose, 5.23% part-time employees, and 3.18% retired or unemployed. In addition, 16% of participants had previously attended a naloxone training session, and just 4.4% of participants regularly carry naloxone on their person.

### Attitudes towards naloxone and overdose

**Fig. 1:**
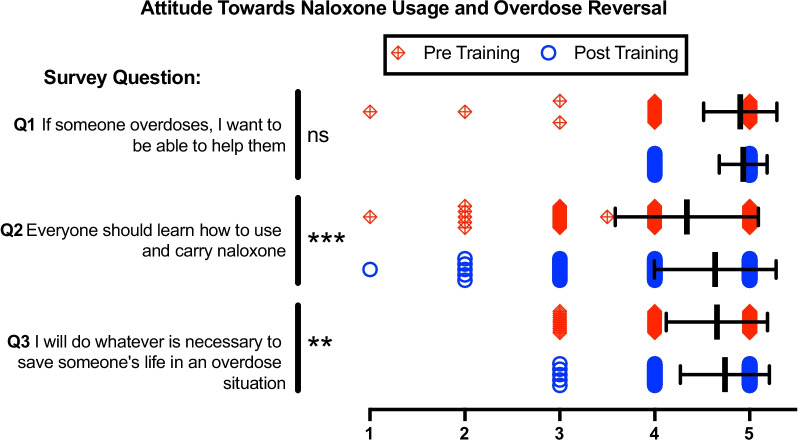
Pre- and post-training survey questions Q1-Q3 characterize attitude towards Naloxone usage and overdose reversal via 5-point Likert scale. 1 = Strongly Disagree, 2 = Disagree, 3 = Indifferent, 4 = Agree, 5 = Strongly Agree. Individual data presented as a scatter dot plot; red diamonds represent pre-training responses while blue circles represent post-training responses. Overlaid black bars indicate Mean ± SD. Q1 *n* = 381, Q2 *n* = 380, Q3 *n* = 380. *p < 0.05, **p < 0.01, ***p < 0.001, and ns = not significant

There was no significant difference in pre- and post- responses in Fig. 1 Q1, although there was a non-significant increase in the post-survey, suggesting increased desire of the participant to help someone who has overdosed. Meanwhile, Q2 and Q3 demonstrated a statistically significant improvement in trainees’ view of naloxone usage and their commitment to helping people experiencing an overdose (Table 2). Overall, these data show that 2/3 questions regarding attitudes of naloxone usage and overdose reversal were improved from the OOART.

### Attitudes towards individuals with OUD

**Fig. 2:**
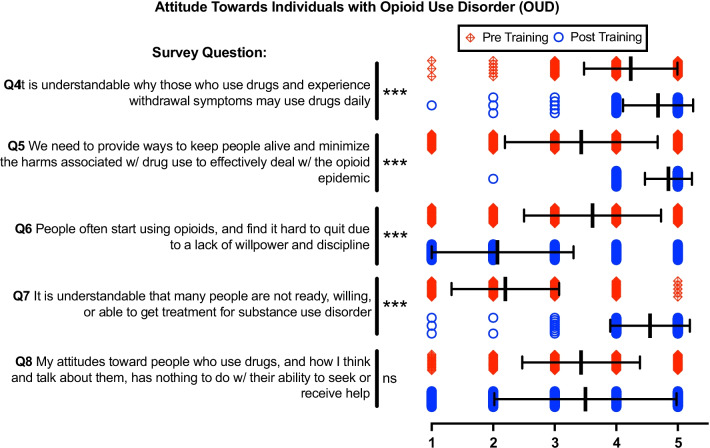
Pre- and post-training survey questions Q4-Q8 characterize attitude towards individuals with Opioid Use Disorder (OUD) via 5-point Likert scale. 1 = Strongly Disagree, 2 = Disagree, 3 = Indifferent, 4 = Agree, 5 = Strongly Agree. Individual data presented as a scatter dot plot; red diamonds represent pre-training responses while blue circles represent post-training responses. Overlaid black bars indicate Mean ± SD. Q4 *n* = 378, Q5 *n* = 369, Q6 *n* = 372, Q7 *n* = 377, Q8 *n* = 369. *p < 0.05, **p < 0.01, ***p < 0.001, and ns = not significant

Figure 2 Q4–Q7 shows statistically significant increases in understanding the nuances, harms, and challenges that people with OUD face. Notably, Q8 had the largest standard deviation in both the pre- and post-survey and was the only question in this category that lacked statistical significance (Table 2)

### Knowledge and self-confidence in handling and overdose

**Fig. 3:**
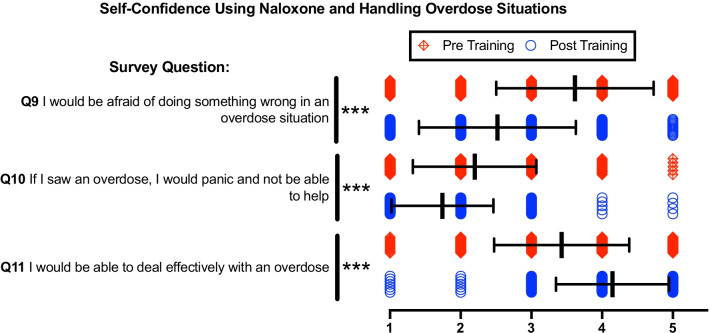
Pre- and post-training survey questions Q9-Q11 characterize attitude towards self-confidence using Naloxone and handling overdose situations via 5-point Likert scale. 1 = Strongly Disagree, 2 = Disagree, 3 = Indifferent, 4 = Agree, 5 = Strongly Agree. Individual data presented as a scatter dot plot; red diamonds represent pre-training responses while blue circles represent post-training responses. Overlaid black bars indicate Mean ± SD. Q9 *n* = 374, Q10 *n* = 379, Q11 *n* = 381. *p < 0.05, **p < 0.01, ***p < 0.001, and ns = not significant

**Fig. 4:**
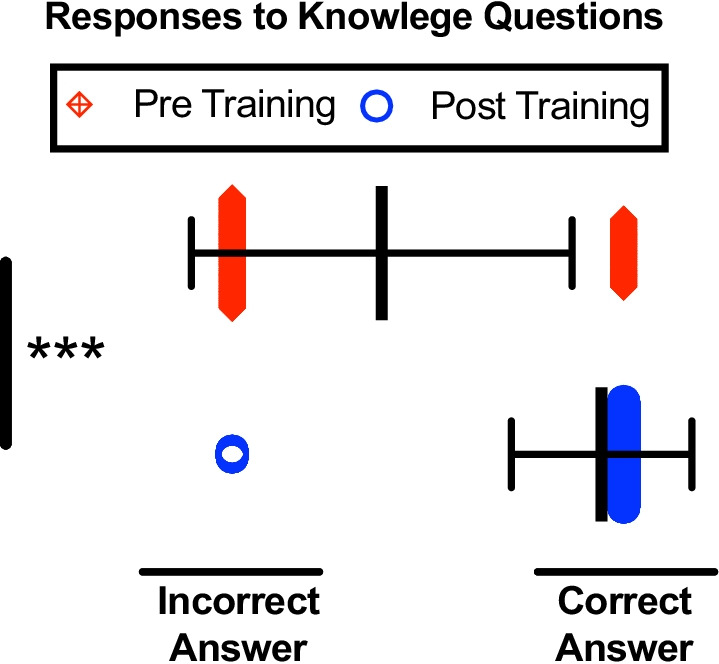
Pre- and post-training knowledge question correctness. Individual data presented as a scatter dot plot; red diamonds represent pre-training responses while blue circles represent post-training responses. Overlaid black bars indicate Mean ± SD. *n* = 984 ***p < 0.001

Figure 3 Q9–Q11 revealed statistically significant increases in trainees’ confidence to respond to and aid an individual experiencing an opioid overdose (Table 2). Figure 4 indicates that the OOART increased the knowledge of how and when to apply naloxone to reverse an opioid overdose event. Participants demonstrated statistically significant improvement in overall percentage correct and in all three individual fact-based questions (Table 2)

## Discussion

The death toll from opioid overdose continues to climb each year in the US, now substantially outnumbering deaths from motor vehicle accidents and other forms of preventable death [2]. Despite the increasing availability of naloxone, only a small fraction of the US population knows how to use this life-saving drug, with few citizens reporting to carry it regularly, as indicated by only 4.4% of participants in this study who described ever carrying naloxone on their person. Given these facts, the current study represents a key step towards increasing the number of individuals versed in the use of naloxone and the quantity of naloxone within the community [13]. Moreover, it provides critical data on one of the largest cohorts to date and, in contrast to the majority of prior studies, evaluated a sample of participants with diverse backgrounds and life experiences.

Aligning with previous reports, this study revealed increases in participant knowledge, indicated by statistically significant improvements in all three fact-based multiple-choice questions, as well as overall percent correct from the pre- to post-survey questions. These findings were in line with participants' perceived increase in competency, demonstrated by statistically significant improvements in Q9–Q11. Comparatively fewer studies have assessed for changes in attitudes towards naloxone and overdose reversal, although those that did have mostly found significant improvements, which also aligns with results from the current study (Fig. 1).

Where our findings diverge from previous studies is in the category relating to attitudes towards individuals with OUD. Specifically, we found significant improvement in four out of five questions pertaining to this category (Fig. 2). After reviewing the responses, we believe the sole question that did not demonstrate improvement, Q8, contained wording that may have confused participants, supported by the lack of change in either direction on post-surveys and its SD being larger than every other question on the survey. Overall, this represents a substantial improvement from our earlier investigation, which was conducted on a cohort composed of only medical students and found statistically significant changes in just one question within the category [33]. Thus, this study, with its larger and more diverse cohort, serves to expand upon our knowledge by demonstrating resounding improvements in attitudes towards individuals with OUD—an outcome that previous OOPPs have been unable to achieve [23–25, 27, 29]. Consistent with this line of logic, we recommend that the traditional OOAS be expanded from accessing ‘readiness’, ‘competence’, and ‘concerns,’ to also include a section for ‘attitudes towards individuals with OUD.’ Such a revision would allow the medical community to gain a more refined perspective on how trainings are working to reduce the public's biases and stigmata against the population suffering from OUD. We believe this is a necessary step in the evolution of the attitudes scale as the content of OOPPs continue to advance.

In addition to our diverse population and large sample size, we surmise that the primary reason we were able to achieve positive outcomes in attitudes towards individuals with OUD was attributable to the unique nature of our innovative OOART. While other OOPPs focus predominantly on protocols for overdose recognition and naloxone administration, a large component of our program describes the sociohistorical forces that have culminated in the opioid epidemic, the biopsychosocial factors driving someone with OUD, and ancillary harm reduction strategies that work further upstream than overdose reversal (Additional file 1: Appendix 1). Of note, our OOART also incorporates an interactive discussion with a person living with OUD. In summary, we extensively address the underpinnings that are responsible for the stigma held towards individuals with OUD, wherein stigma is defined as a process in which people are labeled, stereotyped, and devalued within the context of unequal power relations [38].

In order to truly grasp the inner workings of stigma and OUD, our trainings break down the complexities of their relationship in a manner that is digestible for the general public. By doing so, our goal was to dispel common negative beliefs, biases, and misconceptions about the population. Although colloquial discourse on stigma often discusses it as a single entity, research has shown that when it comes to the opioid epidemic, stigma affects those with OUD on a multitude of levels [38]. Furthermore, one of the most conspicuous variants of stigma discussed throughout our training was public stigma, which occurs when stereotypes about a certain group lead to individuals within said group being perceived as dangerous or guilty of some moral failing, subsequently translating into the general public holding negative attitudes towards them. The impact of public stigma in the case of OUD is indisputable. It was empirically demonstrated by Perry et al. [40], where investigators surveyed 1169 US citizens and found that individuals with OUD experienced a high likelihood of being socially excluded and perceived as untrustworthy, even more so than individuals suffering from schizophrenia and alcohol use disorder. In addition, respondents in that study were more likely to attribute OUD to personal characteristics and less to the environment and/or upbringing. Unfortunately, when people with OUD begin to identify with the public stigma attached to their illness, they can become psychologically distressed and develop maladaptive behaviors that lead to poorer health outcomes. In technical terms, they begin to experience internalized stigma, which has been shown to correlate with decreased quality of life in numerous ways, including struggles in the physical, psychological, and environmental domains [38]. Thus, the transition to internalized stigma is a key step in the ‘Stigma Cycle,’ which is a topic discussed extensively in the OOART training (Additional file 1: Appendix 1). Similar to internalized stigma, individuals with OUD may grapple with anticipated stigma, defined as instances where groups burdened by a stigmatized identity are subjectively aware of such negative attitudes and develop expectations of being rejected [38]. This type of stigma is particularly harmful, given that the fear of being rejected for medication-assisted treatment (MAT) or receiving the label of an “addict” is often cited as a profound determent, if not the most deterring factor in taking initiative to seek help, as noted throughout our trainings [41].

In the case of OUD, public, internalized, and anticipated stigma all intersect to form structural stigma. Reversing this is where our OOART attempts to make the most significant headway. In relation to the healthcare system, structural stigma is pervasive and is most evident in laws regulating the prescription of buprenorphine through the X-waiver. Despite the fact that the partial opioid agonist/antagonist has a strong safety profile and serves as a highly effective treatment for OUD, providers are still mandated to complete additional training before they can obtain the X-waiver and are allowed to prescribe buprenorphine to their patients [42]. Ultimately, this barrier substantially reduces the number of physicians who are permitted to prescribe buprenorphine. For example, there were 985,026 licensed physicians in the USA in 2018 [42]. However, only 56,403 were approved to prescribe buprenorphine. Of those, 72.4% had a 30 patient-limit [43] illustrating the point that even those physicians who want to serve patients with OUD are limited in doing so by current legislative barriers. Moreover, since it is estimated that access to MAT decreases overdose by 50–79% and the risk of recurrence of opioid use by more than 50%, it is imperative that we increase the number of providers capable of prescribing buprenorphine and dismantle the stigmatizing infrastructure impeding its access [40].

To date, OOPPs have succeeded in preventing deaths from overdose with naloxone [44]. However, it is becoming increasingly apparent that by focusing solely on reversal and neglecting to discuss the social implications of OUD, programs may be missing a critical opportunity to educate the public on upstream factors that could facilitate prevention of overdoses before they happen. In consideration of our findings and the above discussion on the ramifications of pervasive stigma, we believe this can be accomplished by designing OOPPs that not only provide training in the use of naloxone but also address the stigmas and legislative regulations responsible for perpetuating the opioid epidemic at a higher level. Furthermore, given the strong improvements in attitudes towards those with OUD found in the current study, efforts should be made to incorporate the unique focus on biopsychosocial and sociohistorical components intrinsic to our OOART into all OOPPs. This will enable trainings to move beyond tertiary prevention of basic naloxone administration and enter into the realm of primary and secondary prevention by addressing systemic impediments that prevail in American culture and society.

### Limitations

Limitations of this study include the lack of long-term follow-up, since post-surveys were only administered immediately following training. In addition, although our cohort was fairly diverse, the majority of participants were full-time students and in their 20 s. It must also be noted that there was an overrepresentation of individuals associated with healthcare related careers. These two limitations may have introduced bias into the sample, as healthcare workers and individuals who voluntarily participated in OOART may be more receptive to learning about overdose reversal and OUD than their peers. Finally, while a considerable number of individuals verbally disclosed their history of OUD or membership in a marginalized group with OUD (i.e. sex workers, individuals affected by HIV, and women who use drugs), we did not collect quantitative data on these variables in the interest of anonymity and maintaining a judgement-free environment. Despite these limitations, the study population remains very diverse compared to similar studies, and the large cohort provided the power necessary to detect small changes in the pre- versus post-study, which may have been a limitation in previous, smaller studies.

## Conclusion and future directions

To improve care of those with OUD, direct educational interventions must be implemented at the level of curricular changes in undergraduate, graduate, and continuing medical education. Other community members, such as police officers, should also be strongly encouraged to attend training sessions. As mentioned, this training should be focused not only on overdose reversal but also harm reduction, stigma, and upstream overdose prevention. We believe this is the best method to transform our society from reactionary prevention of deaths from overdose via naloxone to proactive prevention of overdose by way of systemic change. Future studies are currently underway to determine if changes in knowledge and attitudes persist over time and to quantify the total number of overdoses reversed by participants.

For those wishing to implement curricular changes in their own programs, we suggest starting with the recorded online version of the OOART (Narcan Outreach Project Training) and contacting the authors for further guidance, if necessary.

## Supplementary Information

**Additional file 1: Appendix 1.** Outline of topics discussed in the OOART. The outline is divided into the seven sections that correspond to the PowerPoint presentation. The outline is not all encompassing but covers most topics formally discussed during each training session.

## Data Availability

The datasets used and/or analyzed during the current study are available from the corresponding author on reasonable request.
