# Supplementary material for: Reducing the stigma surrounding opioid use disorder: evaluating an opioid overdose prevention training program applied to a diverse population
Source: Harm Reduct J. 2022 Jan 16;19:5. doi: 10.1186/s12954-022-00589-6 (PMC8761384; doi:10.1186/s12954-022-00589-6)
Supplement: Supplementary file 1 — Additional file 1: Appendix 1. Outline of topics discussed in the OOART. The outline is divided into the seven sections that correspond to the PowerPoint presentation. The outline is not all encompassing but covers most topics formally discussed during each training session. [file 12954_2022_589_MOESM1_ESM.pdf]

# Appendix 1: Outline of Topics Discussed in the OOART

1) ***Section 1: Opioid Basics***
    a. Review of the biological basis of opioids and physiological effects on the body
    b. Overview of different types and formulations of opioids
    c. Description of how naloxone works to negate the effects of opioids
2) ***Section 2: Introduction to the Opioid Epidemic***
    a. Focus on the devastation caused by opioids in Philadelphia (can be adapted to other cities/regions)
    b. How trends in the city and nation demonstrate substantial increases in the number of opioid overdoses and related deaths over the past two decades
    c. Description of the heterogenous distribution of overdoses throughout the city and discussion surrounding why certain areas become hotspots
        i. Statistics depicting how introduction of naloxone in these areas correlated with decreases in lethal overdoses
    d. Explanation of increasing lethality as main drug supplies transitioned from predominance of heroin to fentanyl
3) ***Section 3: A Brief History and the Aftermath***
    a. Provides In depth discussion on the opioid history timeline and the roots of the opioid epidemic
        i. Brief introduction to the origins of opioids and how the drugs themselves evolved over time, starting with opium, then morphine, and finally transitioning to heroin and more potent drugs, such as fentanyl
        ii. Critique of how data was misappropriated from academic journals and incorrectly used to justify the safety of opioids, including NEJM 1980 and Pain 1989
        iii. Implications of pain being deemed "fifth vital sign in 1996
        iv. Purdue Pharmaceuticals capitalizing on earlier events by creating Oxycontin and misrepresenting the risk of severe addiction
    b. Over prescription of pharmaceuticals by the medical community leads to a large number of individuals developing dependence and OUD
        i. Increasing tolerance and high price of pharmaceuticals eventually forces people to transition to the administration of IV drugs that are stronger and cheaper, namely heroin and fentanyl
        ii. The number of deaths from fentanyl skyrockets and becomes the most common cause of drug overdose
4) ***The Experience of Opioid OUD***
    a. Defining and differentiating between the terms: dependence, tolerance, and addiction
        i. Identifying the stigma associated with the word "addiction" and explanation for why OUD/SUD is the preferred terminology
    b. Emphasizing how everyone can be vulnerable to OUD, irrespective of gender, age, race, ethnicity, SES, etc.
    c. Summarizing the multifactorial nature of OUD and describing the biological, psychological, and social forces that can lead to an individual developing and repeatedly relapsing from OUD
    d. Describing how OUD's multifactorial nature makes every case unique, meaning that treatments must be individualized, as there is no "one fits all" treatment
    e. Pointing out the major ways in which stigma may manifest and create barriers to treatment
        i. Blame and moral judgement→ Criminalization → Pathologize → Patronize→Fear and isolation
        ii. Over time, burden of stigma also begins to have a strong effect on family and friends
    f. Clarifying the ways in which the "Stigma Cycle" captures the development and manifestation of stigma
        i. Speculation for how the Stigma Cycle functions in the case of OUD is provided, along with more specific examples that demonstrate the direct impact on individuals with OUD
        ii. Describing how internalization of negative attitudes leads to feelings of shame and learned helplessness
    g. Studies on physicians' attitudes towards individuals with OUD have shown that a majority of healthcare providers hold negative perceptions and are hesitant to accept members of the population as patients
        i. A notable portion of physicians believe that the individual with OUD is responsible for their treatment, often leading to feelings of abandonment and creating a schism between the healthcare system and OUD population
        ii. Studies also show that a large percentage of physicians have never prescribed naloxone and lack knowledge of its significant role in reducing overdose deaths
    h. Explaining how anyone can prevent or reduce stigma and encouraging participants to engage in these strategies

i. Listening to individuals with OUD with compassion and trying to understand their story without passing judgment
ii. Speaking out about injustices, dispelling misconceptions, and disseminating accurate information
iii. Treating people with dignity and viewing them, not as an 'addict, 'junkie; or 'criminal', but as a person, just like you and me
iv. Avoiding language that conveys negative connotations by using neutral language that does not stigmatize (examples provided in presentation)

5) ***Disparities in the Opioid Epidemic***

a. Comparing differences between the crack epidemic of the 80/90's and the current opioid epidemic, with emphasis on how racial and ethnic demographics influence public response and policy making
   i. Example: Implementation of "war on drugs" led to the incarceration of countless African Americans, while incarceration is not the focus of the opioid epidemic, especially in predominately white communities
b. There is significantly less access to treatment centers and MAT in lower income neighborhoods
c. Research shows physicians are less likely to provide narcotics and adequate pain relief to African American patients when compared to their white counterparts

6) ***OUD Treatment and Harm Reduction as a Tool***

a. Identifying what the treatment options are for OUD, with a large portion of the discussion focused on MAT
   i. Differentiating between buprenorphine and methadone by detailing their physiological mechanisms, as well as the pros and cons of each
   ii. Discussing some of the barriers to accessing and receiving MAT such as: insufficient treatment slots, legislative impediments including the mandated X-waiver required for physicians to prescribe buprenorphine, costs, transportation difficulties, comorbidities common to OUD, and social marginalization
b. Defining harm reduction, its philosophy, and its main tenants
   i. Explaining why the implementation of harm reduction strategies is particularly important for individuals who are not ready or unable to seek treatment
   ii. Providing specific examples of harm reduction strategies including needle exchange, safe injections sites, distilled water, fentanyl testing stripes, and more
      1. Followed by information on how anyone can get involved in harm reduction projects
c. Introduction to naloxone's role as a harm reduction tool

7) ***Overdose Reversal, Naloxone Administration, and Post-Reversal Care***

a. Overview of overdose prevention techniques by describing circumstances that increase the risk of overdose and death such as: mixing drugs, using alone, allowing someone else to inject for you, using a new stamp, and a recent hiatus from opioids
b. Providing education on ways to recognize an overdose
   i. identifying its clinical signs and symptoms
   ii. Differentiating between an opioid overdose and overdoses from other drugs
   iii. Comparing what it looks like when someone is overdosed versus just being high
c. Detailing the importance of consent and the principle of implied consent in emergency situations
d. Identifying the chemical and physiological properties of naloxone, as well as possible side effects
e. Overview of the SCARE acronym as a mnemonic for the overdose reversal process
   i. S: Stimulate; C: Call 911/clear the areas; A: Administer naloxone; R: Rescue Breathes; E: Evaluate
f. Simulating an overdose situation with emphasis on the 5 steps of SCARE
g. Discussing key components of post-reversal care such as: recovery position, expectation of precipitated withdrawal, importance of going to the hospital, risk of overdosing again with repeated use given that some opioids may stay in the system longer the naloxone, and emphasis on compassion and understanding
h. Clarifying intricacies and legal liabilities, with focus on the "Good Samaritan Law" and its exceptions
i. Bringing awareness to the "Standing Order," accompanied by information on where/how to obtain naloxone
